# Framing natural assets for advancing sustainability research: translating different perspectives into actions

**DOI:** 10.1007/s11625-018-0599-5

**Published:** 2018-07-17

**Authors:** Maria Jose Martinez-Harms, Stefan Gelcich, Rainer M. Krug, Fleur J. F. Maseyk, Hannah Moersberger, Archi Rastogi, Geoffrey Wambugu, Cornelia B. Krug, Eva M. Spehn, Unai Pascual

**Affiliations:** 1 Center for Applied Ecology and Sustainability (CAPES), Center for the Study of Multiple-Drivers on Marine Socio-Ecological Systems, Pontificia Universidad Catolica de Chile, Avd. Libertador Bernardo O’Higgins 340, Santiago, Chile; 2 Australian Research Council Centre of Excellence for Environmental Decisions, School of Biological Sciences, The University of Queensland, Saint Lucia, QLD 4072 Australia; 3 Department of Evolutionary Biology and Environmental Studies, University of Zurich, Winterthurerstrasse 190, 8057 Zurich, Switzerland; 4 The Catalyst Group, PO Box 362, Palmerston North, 4440 New Zealand; 5 Future Earth Paris Hub, Centre National de la Recherche Scientifique (CNRS), 4 Place Jussieu, 75005 Paris, France; 6 Universalia Management Group, 245 Victoria Avenue, Suite 200, Westmount, QC Canada; 7 School of Natural Resources and Environmental Studies, Karatina University, PO Box 1957-10101, Karatina, Kenya; 8 URPP Global Change and Biodiversity, University of Zurich, Winterthurerstrasse 190, 8057 Zurich, Switzerland; 9 Global Mountain Biodiversity Assessment, Institute of Plant Sciences, University of Bern, Altenbergrain 21, 3013 Bern, Switzerland; 10 Basque Centre for Climate Change, University of the Basque Country (UPV-EHU), Sede Building 1, 1st Floor, Scientific Campus, Leioa, 48940 Bilbao, Spain; 11 Ikerbasque, Basque Foundation for Science, María Díaz Haro, 3, 48013 Bilbao, Spain; 12 bioDISCOVERY, Department of Geography, University of Zurich, Winterthurerstrasse 190, 8057 Zurich, Switzerland; 13 Centre for Development and Environment, University of Bern, Mittelstrasse 43, 3012 Bern, Switzerland

**Keywords:** Sustainability, Knowledge exchange, Ecosystem services, Natural capital, Human actions

## Abstract

Sustainability is a key challenge for humanity in the context of complex and unprecedented global changes. Future Earth, an international research initiative aiming to advance global sustainability science, has recently launched knowledge–action networks (KANs) as mechanisms for delivering its research strategy. The research initiative is currently developing a KAN on “natural assets” to facilitate and enable action-oriented research and synthesis towards natural assets sustainability. ‘Natural assets’ has been adopted by Future Earth as an umbrella term aiming to translate and bridge across different knowledge systems and different perspectives on peoples’ relationships with nature. In this paper, we clarify the framing of Future Earth around natural assets emphasizing the recognition on pluralism and identifying the challenges of translating different visions about the role of natural assets, including via policy formulation, for local to global sustainability challenges. This understanding will be useful to develop inter-and transdisciplinary solutions for human–environmental problems by (i) embracing richer collaborative decision processes and building bridges across different perspectives; (ii) giving emphasis on the interactions between biophysical and socioeconomic drivers affecting the future trends of investments and disinvestments in natural assets; and (iii) focusing on social equity, power relationships for effective application of the natural assets approach. This understanding also intends to inform the scope of the natural asset KAN’s research agenda to mobilize the translation of research into co-designed action for sustainability.

**Electronic supplementary material:**

The online version of this article (10.1007/s11625-018-0599-5) contains supplementary material, which is available to authorized users.

## Introduction

Global efforts to achieve the United Nations sustainable development goals (SDGs) require an understanding of how nature and biodiversity will be impacted by global environmental change. Many natural systems are being pushed beyond their limits (Rockström et al. 2009), as the ability to produce socially desired goods in the short term is favoured over critical longer-term ecosystem processes (Rasmussen et al. 2018). Consequently, signs of escalating and compounded stresses are evident at global, national and local scales and are reflected in local and regional scarcities of water, widespread land degradation and loss of biodiversity (Griggs et al. 2013; IPBES 2018). The consequences of biodiversity loss for ecosystem functioning, the provision of an array of regulating ecosystem services, and ultimately for human well-being have been identified as a major concern amongst the scientific community (Hooper et al. 2005; Balvanera et al. 2006; Díaz et al. 2006; Worm et al. 2006; Cardinale et al. 2012; Rasmussen et al. 2018).

The sister twin “natural capital” and “ecosystem services” approach, putting emphasis on the multidimensional analysis of the benefits provided by ecosystems, has gained increasing attention in some policy circles and business in the last 20 years (Costanza et al. 2017). This is mainly because it allows considering these benefits in decisions from which they were usually absent (Maes et al. 2012; Bennett 2016). This has the potential to result in decision-making processes that take into account the benefits that nature provides to people facilitating communication and collaboration among scientists, practitioners, decision-makers, and other stakeholders. Ecosystem services science has experienced great popularity and advances (De Groot et al. 2010; Bennett and Chaplin-Kramer 2016) with several high profile and referenced efforts (MA 2005; Sukhdev 2010). While the importance of natural capital (i.e. the stock of natural resources) and ecosystem services is increasingly being recognized (Dasgupta 2010; Dominati et al. 2010; Kareiva 2011; Bateman et al. 2013; Guerry et al. 2015; Maseyk et al. 2017), there has been a relatively modest uptake of these advances in decision-making (Laurans et al. 2013; Martinez-Harms et al. 2015) and practical guidance on taking responsibility and actions for management are still lacking.

In a recent contribution, Díaz et al. (2018) presented the notion of “Nature’s Contributions to People” (NCP) as a central element of the Intergovernmental Science-Policy Platform on Biodiversity and Ecosystem Services’ (IPBES) conceptual framework. The authors proposed NCP as a broader framing building on the ecosystem services approach while opening up to other perspectives, mostly associated with the social sciences and humanities, which are rich in explaining the complex and diverse realities about people’s relationships with nature. The NCP approach has triggered a lively debate with some players in the ecosystem services science community questioning whether a paradigm shift or drift from ecosystem services to NCP is justified (e.g. Maes et al. 2018; Peterson et al. 2018; Faith 2018). We believe that the diversity of perspectives across scientific disciplines enriches and facilitates progress in science, without inhibiting policy making. To foster support to the IPBES process, we agree that there is no one-size-fits all framework to cover all the diverse problems that nature and people face (Peterson et al. 2018) and that the attempt by the NCP approach to enlarge the tent which can advance integrating a growing knowledge base and the need for transformative action towards sustainability. The NCP approach can help to find a flexible and adaptive approach according to the specific policy process objectives and decision-making audience, facilitating collaboration and knowledge exchange among different stakeholders connecting knowledge and action.

Future Earth, a global network for sustainability science, has recently launched a new global initiative, the natural assets knowledge–action network (KAN), which directly connects to IPBES. Similar to the broadening of the ecosystem services approach by the framing of NCP, a new flexible and adaptive framing has recently been adopted by Future Earth based on the notion of ‘natural assets’. As with NCP, the aim is also to translate and bridge among different knowledge systems and different perspectives about people’s relationships with nature. The natural assets approach emphasizes the role of human actions on reshaping nature and can complement the NCP framework. Emphasizing on human actions to protect or responsibly manage nature can help connect knowledge systems and actors engaged in reshaping nature. To operationalize the natural assets approach, the KAN brings together scientists and other stakeholders from a wide variety of disciplines, sectors and organizations with the ambitious challenge of creating a community of practice for achieving sustainable stewardship of natural assets underpinning human well-being. Activities within the natural assets KAN strive to respond and shape nature under conditions of uncertainty and change.

This paper aims to clarify the natural assets concept for the global Natural Assets KAN community discussing challenges that the KAN will face in operationalizing the natural assets concept. These challenges are:

(i) embracing richer collaborative decision processes to build bridges between different human-nature perspectives;
(ii) Emphasis on the interactions between biophysical and socioeconomic drivers affecting the future of natural assets; and
(iii) focusing on social equity, power relationships and discourses for effective application of the natural assets approach.

Addressing these challenges will be useful to inform the scope and definition of objectives, and ensure the relevance of the activities of the natural assets KAN.

## Concepts underlying the natural assets approach: natural capital, ecosystem services and nature’s contributions to people

There has been a boom of interest in writing and reading about nature as people seek to reconnect with ecosystems. This explains the increment of concepts and notions (e.g. natural assets, natural capital, natural capital stocks, ecosystem services, nature’s contributions to people, natural-based solutions) and the literature to explain human–nature relationships attached to efforts to conserve and manage ecosystems (see Table 1).

**Table 1:** Definitions of the most common terms used to explain people’s relationships with nature appearing in the literature

Terms	Definitions	Links
Natural assets (NA)	Biotic and abiotic components that are owned and managed leading to the flow of ecosystem services over time (Mace et al. 2015)	NA = N = E
Natural capital (NC)	The abiotic and biotic elements of nature, including all natural resources (such as soil, water, vegetation, species) and physical, biological, and chemical processes (Mace et al. 2015)	NA → NC
Natural capital stocks (NCS)	Natural capital consists of stocks of natural assets—the amount of a material in a given pool, form, or state in an ecosystem (Mace et al. 2012) that yield a flow of valuable ecosystem goods or services into the future (Costanza and Daly 1992)	NA → NC/NCS
Nature (N)	Natural world with an emphasis on the diversity of living organisms and their interactions among themselves and with their environment (Díaz et al. 2015)	NA = N=E
Ecosystem (E)	A dynamic complex of plant, animal, and microorganism communities and their non-living environment interacting as a functional unit (MA 2005)	E → BD
Biodiversity (BD)	The variability amongst the different levels (ecosystem, species, genes) of ecological organization including living organisms from all sources such as inter alia, terrestrial, marin,e and other aquatic ecosystems, and the ecological complexes of which they are part (CBD 2010; Mace et al. 2012)	E → BD → EP → ES
Ecosystem functions and processes (EP)	An interaction among organisms; ecological processes frequently regulate the dynamics of ecosystems and the structure and dynamics of biological communities (Mace et al. 2012)	E → BD → EP → ES
Nature contributions to people (NCP)	“All the contributions, both positive and negative, of living nature (diversity of organisms, ecosystems, and their associated ecological and evolutionary processes) to people’s quality of life” (Díaz et al. 2018)	NCP → ES
Ecosystem services (ES)	Benefits that flow from natural capital to society (Boyd and Banzhaf 2007; Guerry et al. 2015)	ES → B
Flows (F)	It is the realization of an ecosystem service to people (Mitchell et al. 2015)	E → B
Benefits (B)	The ways in which ecosystems improve human well-being through the provision of ecosystem services (Mitchell et al. 2015)	ES → B→V
Values (V)	“Values can refer to a principle associated with a given worldview or cultural context, a preference someone has for a particular state of the world, the importance of something for itself or for others, or simply a measure” (Pascual et al. 2017)	V → NCP → A
Nature-based solutions (NBS)	Concept to promote nature as a means for providing solutions to climate mitigation and adaptation, food security, social and economic development (Nesshöver et al. 2017)	N ← NBS
Ecological infrastructure (EI)	Landscape elements, ecosystems, ecosystem services, and the interconnections within and between them (Bristow et al. 2010)	N ← EI
Governance (G)	Describes how the process of management decisions are made or the development of how policies and strategies may be constructed (Díaz et al. 2015)	NA ← A←G
Human actions (A)	Principles, rules, and guidelines designed to influence and determine all major decisions	NA ← A←G

The links column indicates how the authors of this publication interpret the relationship between the concepts, indicating if they are interchangeable (=) or whether the concepts are interrelated but not the same (→)

**Fig. 1:**
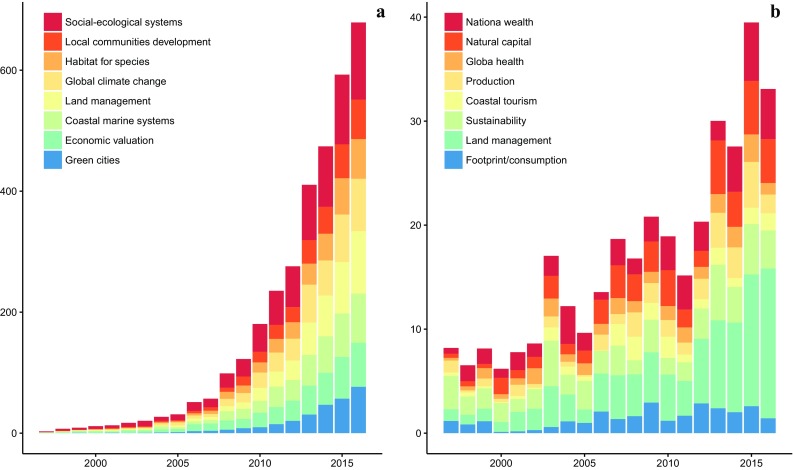
Research available in the ISI Web of Knowledge identifying the number of publications within the eight most frequently researched topics on ecosystem services representing a sample of ~ 4000 papers (**a**) and natural capital representing a sample of ~ 350 papers (**b**), derived using a topic-modelling approach (https://code.google.com/archive/p/topic-modeling-tool/) (see supplementary material for detailed methods)

Among the diverse terminology, the twin stock-flow sisters “natural capital” and “ecosystem services” have been the most popular ones during the last two decades. For example, between 1997 and 2016 there have been more than 13,500 peer-reviewed publications containing the term “ecosystem services” in the ISI Web of Science and 910 for “natural capital” (see Supplementary Material for detailed description of topic analysis tool). Ecosystem service research has predominately been focused on the topics related to social–ecological systems, local development, land/sea management, and global change scenarios, among other topics (see Fig. 1a). While there are similarities like the recurrent management aspect, the natural capital term has been mainly focused on wealth, assets and production landscapes (see Fig. 1b). The ecosystem services research timeline clearly shows the rapid increase in papers published since the emergence of the concept with the publication of Nature’s Services (Daily 1997) and the Millennium Ecosystem Assessment (MA 2005).

### Natural capital

Natural capital is the “stock” from which useful ecosystem goods and services can flow to people, comparable conceptually to the stock of human or financial capital (Costanza et al. 1997; Akerman 2003; Gómez-Baggethun and De Groot 2010; Mace et al. 2015; Maseyk et al. 2017). Natural capital comprises all abiotic and biotic elements as well as ecosystems and within ecosystems biodiversity (Mace et al. 2015). There is plenty of scientific evidence linking biodiversity to ecosystem functioning and their effects on ecosystem services provision (Balvanera et al. 2006; Cardinale et al. 2012; Mace et al. 2012; Duncan et al. 2015). Ecosystem functioning depends on biodiversity and changes in its composition, abundance and function could change the structure of ecosystems affecting the flow of ecosystem services to society (Balvanera et al. 2006; Mace et al. 2012). The links between biodiversity and ecosystem service provision are still not sufficiently well known to predict the consequences of biodiversity changes (Harrison et al. 2014); however, there is evidence that a decline in biodiversity limits the provision of some ecosystem services in favour of others, which is relevant for management (Cardinale et al. 2012).

The characteristics of ecosystems and landscapes, such as species composition, land cover, climatic conditions, and landscape configuration modulate the nature and magnitude of ecosystem services that flow from the natural capital to societies. Societies are deeply embedded within ecosystems, depending on them for survival, while simultaneously creating both positive and negative impacts on them. While many of the benefits that flow from natural capital can be enhanced with technology and engineering, they cannot be replaced (Mace et al. 2015). For natural capital to contribute to human well-being and the provision of ecosystem services, there is need for some input of human capital in the form of management interventions.

### Ecosystem services

Ecosystem services are the benefit flows from natural capital to society. The provision of ecosystem services is supported by the relationships between natural capital and the distribution of people in the landscape, as well as those management actions that modulate access to ecosystem services. Three types of ecosystem services can be distinguished (MA 2005). Provisioning services are directly taken out and consumed from ecosystems and can often be quantified and valued in economic terms such as clean water, raw materials like timber and fibres, and food production among others. Regulating services are those acting as regulators of ecosystem processes such as climate regulation, erosion control, flood regulation, and soil waste assimilation among others. Cultural services are the tangible and intangible benefits that result from human relations with the natural environment (Chan et al. 2012), for example: nature-based tourism and recreation, natural heritage, inspiration, scenic beauty, and many other relational values.

### Nature’s contributions to people

The nature’s contributions to people (NCP) approach by IPBES (Pascual et al. 2017; Díaz et al. 2018) allows considering nature as an asset, but it also goes beyond regarding nature as a stock of resources. A generalizing perspective, similar in spirit to the ecosystem services approach, and a context-specific perspective that allows other than a stock-flow relationship with nature implies that the values of NCP embrace a diversity of worldviews across cultures and in so doing recognizes value pluralism (Pascual et al. 2017). For example, relational values, defined as the importance of nature in fostering desirable relationships between people and nature (Chan et al. 2016), are an important component of IPBES’ inclusive valuation of NCP (Pascual et al. 2017). Such inclusive valuation stems from the realization that the benefits and detriments to humans from natural assets are linked to well-being in diverse and manifold ways. For example, the benefits derived from NCP contribute to changes in living standards, nutritional status, mortality rates, equity and social conflicts, security in the face of extreme environmental conditions, or happiness. Values are differentially perceived either as costs (detriments from nature) or benefits (positive contributions) by individuals and societies (van Oudenhoven et al. 2012; Pascual et al. 2017).

### Framing human well-being

Numerous frameworks linking human well-being with natural capital and the provision of ecosystem services have been developed during these last two decades and are rapidly evolving (MA 2005; Boyd and Banzhaf 2007; De Groot et al. 2010; Dominati et al. 2010; Haines-Young and Potschin 2010; Sukhdev 2010; Tallis et al. 2012; van Oudenhoven et al. 2012; Díaz et al. 2015; Maseyk et al. 2017).The focus of the frameworks has been on understanding the mechanisms behind the delivery of ecosystem services. The delivery of ecosystem services depends on the capacity of the ecosystem to provide a service (supply), on the anthropogenic and natural stressors influencing ecosystem service delivery (ecological pressures), the amount of the service required by society (demand), and the realization of a service experienced by people (flow) (Haines-Young and Potschin 2010; Tallis et al. 2012; Villamagna et al. 2013; Mitchell et al. 2015).

Recent frameworks address ecosystem service assessments from the supply to the demand side, covering three value domains of ecosystem services: biophysical, sociocultural, and monetary (Martín-López et al. 2014). For example, the supply side addresses the domain of biophysical value representing ecosystem service potential delivery, while the demand side refers to benefits to human well-being that have a sociocultural and/or monetary value (Martín-López et al. 2014).

The conceptual framework of the IPBES proposes three basic elements constituting a human–environmental system operating at different temporal and spatial scales: (a) nature (the natural environment with its diversity of living organisms—adding to this evolutionary processes and embracing other world views), (b) NCP, and (c) a good quality of life (Díaz et al. 2015, 2018). IPBES is launching (in 2018) an assessment on the inclusive valuation of NCP for decision-making which is targeted at science-policy initiatives highlighting a pluralistic approach to recognize the multiple values that different stakeholder groups hold on NCP (Pascual et al. 2017).

While the breadth of approaches to describe the provision of ecosystem services from natural capital has facilitated progress in sustainability research, the most critical questions posed by decision-makers in the realm of sustainability have not yet been answered (Villamagna et al. 2013; Bennett and Chaplin-Kramer 2016). For example, why has research that underlies environmental policy agendas (e.g. SDGs, Aichi targets) not always been effectively translated into practice? Where in a human–environmental system should we intervene to change its overall behaviour? A critical limitation to implementing a natural assets approach for decision-making is that existing frameworks lack explicit reference to human actions (Mace et al. 2015; Maseyk et al. 2017). To provide informed management interventions, it needs to be clarified how the provision of ecosystem services is underpinned by the complex interactions between ecological and human dimensions.

## Natural assets: contributing to an inclusive framing on people’s relationships with nature

The way society interacts with and perceives nature shapes many of the paradigms underpinning human–environmental systems (e.g. ecosystem services, natural capital, NCP). The functioning of a system partly depends on the degree to which people’s dependency of nature is acknowledged, and the extent to which human–nature relationships are identified as essential to human well-being. In a recent publication, people’s relationships with nature and their impacts on conservation and management outcomes were identified as a pathway in which transformational change towards sustainability can be leveraged (Abson et al. 2017). Moreover, the implementation of the natural assets approach is very timely, as several initiatives (Convention on Biological Diversity Aichi Targets, CBD (2010); The Economics of Ecosystems and Biodiversity TEEB (Sukhdev 2010); and The Intergovernmental Science-Policy Platform on Biodiversity and Ecosystem Services, IPBES (Perrings et al. 2011) are focusing attention on the recognition of human–nature relationships for human livelihoods and a good quality of life. The implementation challenge is to turn this recognition into policies and decisions that can guide the wise management of nature. The natural assets approach could play a key role by emphasizing the role of human actions aiming to connect knowledge systems and actors engaged in reshaping nature.

Human–nature relationships are also moral and ethical obligations that govern appropriate human actions towards the environment (Abson et al. 2017). Human actions influence the condition of natural assets influencing the provision of ecosystem services (Dominati et al. 2010; Palomo et al. 2016, Maseyk et al. 2017). This is illustrative of the need to embed both the social and ecological dimensions of the natural assets approach in policy making. Focusing on changes in condition (quality and quantity) of natural assets allows for an understanding of the impact of policy outcomes on natural assets. Policy processes provide pre-conditions, limitations, and motivations for human actions.

Natural assets have been previously defined as the components of natural capital that can be owned or managed, for example, ecological communities, minerals, freshwaters, land, the atmosphere, coasts, as well as the natural processes and functions that underpin their operation (Mace et al. 2015). Here, natural assets are defined as an umbrella term aiming to translate and bridge among different knowledge systems and different perspectives on people’s relationships with nature. The natural assets approach embraces the need for richer processes of knowledge exchange among different perspectives on peoples’ relationship with nature, ranging from the production of knowledge to the transformation of knowledge into actions (see Table 2).

**Table 2:** Definitions of the different knowledge process stages since its production to its transformation

Knowledge process stage	Definition
Knowledge production	New knowledge produced as an output of a process either in isolation or co-created through participation and engagement with knowledge users (Berkes 2009; Fazey et al. 2013)
Knowledge transfer	One-way process implying linear delivery and reception of knowledge (Fazey et al. 2013)
Co-production of knowledge	It is a collaboration process aiming to bring together a diversity of knowledge systems to address a defined problem and build an integrated understanding of that problem (Armitage et al. 2011)
Knowledge exchange	Multiple path knowledge process implying multiple delivery and reception of knowledge with mutual benefits and mutual learning (Fazey et al. 2013; Reed et al. 2014; Nguyen et al. 2017)
Knowledge mobilization	Multiple path knowledge process of linking scientists, decision-makers, and practitioners to improve the use of knowledge in practice (Edelstein 2016)
Sharing knowledge	Multiple path knowledge process implying multiple delivery and reception of knowledge with mutual benefits and mutual learning with greater recognition of the value of the knowledge of those sharing the knowledge (Fazey et al. 2013)
Knowledge translation	Implies communication of knowledge using a language modified for knowledge actors (Fazey et al. 2013)
Knowledge systems	Networks of agents, practices, and institutions that organize the production, transfer, and use of knowledge (Peterson et al. 2018)
Knowledge actors	Individual players involved in the exchange and mobilization of knowledge (knowledge producers, intermediaries and users) (Reed et al. 2014)
Knowledge-action	Outcome of the knowledge expressed in change of practices (Nguyen et al. 2017)
Knowledge transformation	Changing the knowledge towards a different state or condition through its internalization as social constructions. (Fazey et al. 2013; Abson et al. 2017)

The key is bridging strategically across multiple knowledge–action interfaces to ensure relevancy across a diversity of perspectives and values. Building natural assets knowledge for sustainability requires approaches that can cope with pluralism and link different knowledge systems (Clark et al. 2016; Peterson et al. 2018) while respecting the integrity of each knowledge system.

## An agenda for natural assets research

This section discusses some key areas that Future Earth will need to tackle in its aim to bridge knowledge and action with regard to sustainability through a natural assets lens. These areas are associated with various challenges and current knowledge gaps identified in the sustainability science literature (e.g. Martinez-Harms et al. 2015; Bennett 2016; Rose et al. 2017, 2018; Pascual et al. 2014; Berbés-Blázquez et al. 2016).

### Embrace richer collaborative decision processes

Despite increasing awareness of the need for evidence-based decision-making (Sutherland et al. 2004; Sutherland and Burgman 2015; Tengo et al. 2017), current research has failed to inform practice as intended (Knight et al. 2006; Cowling et al. 2008; Toomey et al. 2017). The gap between the knowledge generated by scientists and its uptake by policy and actions is a widely recognized challenge in applied ecology (Hulme 2014), conservation (Arlettaz et al. 2010; Toomey et al. 2017), and ecosystem services science (Cowling et al. 2008). Numerous researchers have highlighted the urgent need to narrow the gap between sustainability science and its application in decision-making (Knight et al. 2006; Cowling et al. 2008; Toomey et al. 2017). Despite some advances (Gelcich et al. 2010; Ruckelshaus et al. 2015), further progress is required as translating knowledge into practice change is fraught with difficulties, and several challenges remain that create barriers which prevent narrowing the gap further (Abson et al. 2017). A key issue is to identify spaces of agreement and be able to collaboratively engage with problems faced by policy-makers (Oldekop et al. 2016). Any bid to inform decision-making requires research to be inspirational and useful for end users, be responsive to stakeholder needs from the outset, and ensure collaboration with practitioners both before research initiation, during the research process, and after its completion (Cowling et al. 2008; Gelcich et al. 2010; Martinez-Harms et al. 2015). Advancement requires both scientists and practitioners to participate in a reciprocal and frequent exchange of information and knowledge (Hulme 2014). The field of knowledge exchange (Dunlop 2014; Jordan and Russel 2014) can help to embrace the complexity of translating different knowledge systems (Verburg et al. 2015) and seek to address the constraints that might limit effective knowledge transfer (Scarlett 2013).

Future Earth has adopted the core principle of knowledge co-production (see Table 2), and this will be particularly relevant in narrowing the gap between the implementation of the natural assets approach in decision-making (Reyers et al. 2015). The knowledge co-production approach is a collaborative process to respond to complex problems by bringing together different types of knowledge and creating an integrated understanding of those issues (Armitage et al. 2011). The principle of co-production is presented as the most innovative aspect of Future Earth and is the key attribute distinguishing the research programme from existing initiatives (van der Hel 2016). The knowledge co-production approach enables collaboration between stakeholders with different visions of the role of science to human well-being. For example, Reyers et al. (2015) applied and assessed a knowledge co-production approach with beneficiaries and managers of natural assets and found the approach to be successful in generating shared knowledge and knowledge–action outcomes for sustainability (see Table 2 for definitions). Participatory approaches may have the potential to better provide evidence for local interests and requirements for natural assets (Dunlop 2014); empower stakeholders to act locally (Armitage et al. 2011); enable sustainable transformations; and improve land governance through collective decisions on natural assets (Gelcich et al. 2010; Verburg et al. 2015).

A recent comprehensive review (Martinez-Harms et al. 2015) found that very few studies on ecosystem services management have adequately dealt with implementing evidence-based decisions. If the research supported by the Natural Assets KAN aims to better link knowledge to real-world actions and outcomes, it must consider transparent objectives, seek to evaluate the consequences of alternative management actions, and facilitate closer engagement between science and practice. Evidence-based knowledge should underpin management decisions for natural assets, and these decisions should account for the multiple values and preferences of stakeholders.

**Fig. 2:**
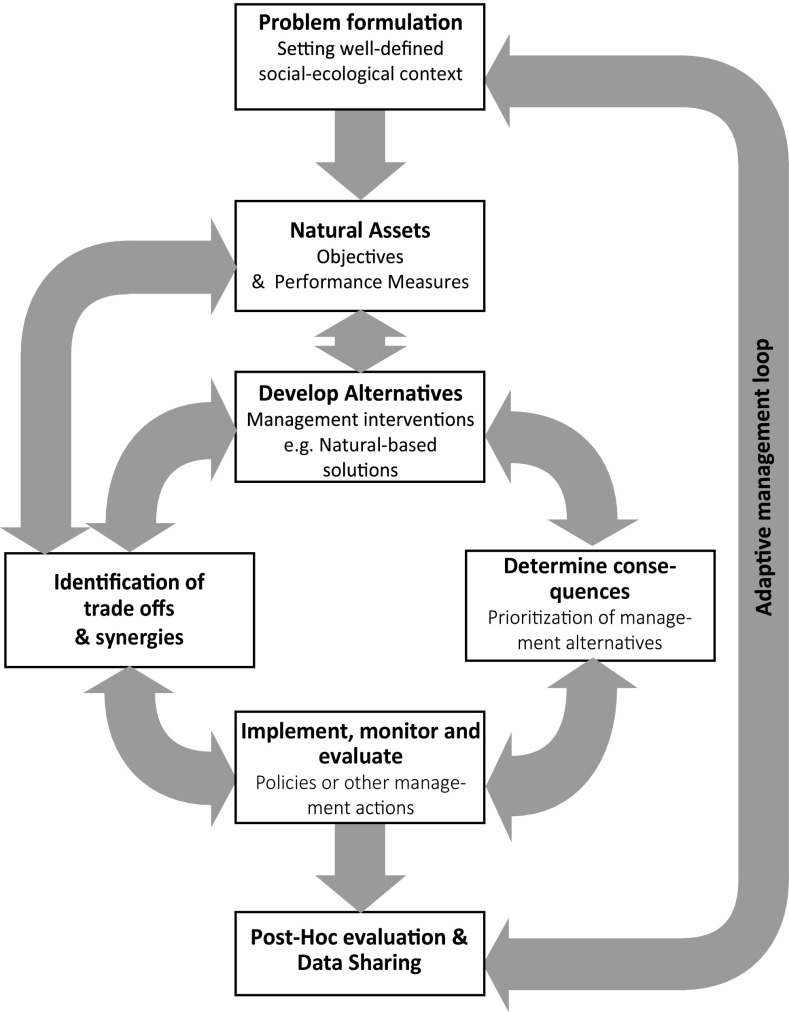
Example of one of the decision support tools to connect knowledge into action. The structured decision-making process represents a flowchart outlining decisions on natural assets. The figure represents a semi-dynamic process starting with the problem formulation and defining a well-defined social–ecological context followed by setting transparent objectives that are those natural assets elements relevant for the study context and the performance measures to test those objectives. The following stages are the dynamic part of the process (setting management alternatives and scenarios, assessment of trade-offs between potential management alternatives, prioritization of alternatives and the implementation of polices), in which one could link any of these stages at any direction. The arrow connecting the trade-offs with the objectives means a decision-maker’s value with respect to multiple objectives. Adaptive management is presented as the overarching cyclical pattern, such that the final stage cycle back to the problem formulation stage based on the outcome of the previous cycle. Adapted from Gregory et al. (2012)

As natural assets management takes place in complex, uncertain, and dynamic social–ecological contexts (Folke et al. 2005), there is increasing attention towards better methods for linking knowledge to action (Schwartz et al. 2017). Decision support tools like structured decision-making (Bower et al. 2017), systematic mapping (Dicks et al. 2014), and the multiple evidence approach (Tengö et al. 2014, 2017) offer a set of responses to this challenge. The structured decision-making framework (Gregory et al. 2012) offers an avenue for making better evidence-based decisions, emphasizing the need for proper problem consideration and formulation and including steps for monitoring and evaluation (Bower et al. 2017) (see Fig. 2). Recently, Bower et al. (2017) recommended the implementation of clear and documented structured decision-making processes and archiving results in a global database to support environmental professionals in making future evidence-based decisions. This solution aims to improve knowledge–action outcomes (see Table 2), enhancing transparency and information sharing.

Systematic mapping is a rigorous technique used to synthesize the state of knowledge for a question or topic, giving a reliable overview of the breadth of science often needed for policy-based questions. On the other hand, the multiple evidence base approach aims to connect and bridge among different epistemologies, producing inclusive understandings that can be used as a starting point for collaborative problem formulation and knowledge co-production (Tengö et al. 2014, 2017). These approaches go beyond just focusing on the quantification of natural assets and instead look through the lens of the whole decision-making process, starting with understanding the human–environmental context with a focus on representing the concerns and aspirations of multiple knowledge systems (Runge et al. 2011; Gregory et al. 2012).

### Focus the decision process on the development of scenarios that capture interactions between human and ecological dimensions of natural assets

Natural assets management deals with high uncertainty due to constant changes in socioeconomic trends, environmental conditions, and social values (Brunner et al. 2017). The scenarios are powerful mechanisms to explore possible outcomes for the future of natural assets due to multiple pathways of future human development, thus explicitly incorporating uncertainty (Rosa et al. 2017). Currently, however, the majority of scenario applications have been targeted to explore the effects of humans on ecosystems, ignoring the role of ecosystems underpinning development and human well-being (Cavender-Bares et al. 2015; Rosa et al. 2017). The next generation of scenarios supported by Future Earth should focus on targets for human development. This is particularly important for achieving the United Nations’ sustainable development goals (SDG), as human development targets within these goals are increasingly connected with targets for nature (Rosa et al. 2017). Future scenarios should focus on the potential synergies and trade-offs between ecosystem services, as well as maintaining or enhancing natural capital to generate future services (Cavender-Bares et al. 2015), and should also address social–ecological feedbacks that are critical for anticipating regime shifts (Bauch et al. 2016). The IPBES considers scenarios as a key tool to assess policy outcomes on nature and nature contributions to people (Díaz et al. 2015; Kok et al. 2016). However, to improve the policy relevance of future IPBES scenarios, the panel needs to engage with the great diversity of local contexts through transdisciplinary approaches, integrating multiple sectors, and linking local to global scale contexts (Kok et al. 2016). Future Earth is a critical contributor to helping IPBES achieve this target, e.g. through mobilizing stakeholder communities and through providing expertise on the co-production of transformative scenarios.

Reporting uncertainty and assessing the robustness of potential outcomes are also critical for ensuring the production of quality scenarios and for delivering credible conclusions (Hamel and Bryant 2017). To identify areas that require management interventions, it is critical to quantify and locate where these uncertainties occur. Nonetheless, there has been a poor uptake of uncertainty analyses within scenarios describing ecosystem service provision (Hamel and Bryant 2017). Most studies dealing with scenarios have several limitations in capturing all the different possible sources of uncertainty and modelling future outcomes that warrant consideration. Scenarios rarely consider emergent properties, complexities, interconnections, and synergistic interactions among the multiple drivers of change and ecosystem services (Liu et al. 2015).

Recently, Hamel and Bryant (2017) and Milner-Gulland and Shea (2017) summarized the commonly perceived challenges for addressing uncertainty analysis in ecosystem services assessments and ecological studies. These include: avoiding uncertainty because it is too difficult and takes time, focusing on trivial uncertainties, and allowing scarce and poorly characterized data to create too much uncertainty which in turn makes it difficult to assess and communicate uncertainty. Substantial knowledge of relevance to natural assets already exists in other fields (e.g. climatology, hydrology, integrated assessment) to address the uncertainty that can be directly transferred to natural assets and help inform more credible decisions (Henrichs et al. 2010; Milner-Gulland and Shea 2017).

Existing models could be improved with more finely-tuned parameters under future conditions, as natural assets are likely to vary across landscapes and seascapes according to biophysical and socioeconomic parameters. Models that couple social and ecological dynamics require the use of powerful decision support tools (e.g. Markov decision-making, supply chain analysis, multilevel modelling, agent-based modelling) to be able to predict the emergence of unexpected drivers of change (Liu et al. 2015). Agent-based models, for example, can be very useful to model human well-being in different scenarios and to model individual’s motivations that can impact the different possible pathways of global change drivers (Rosa et al. 2017).

When resources or modelling expertise is not available for managers, even the simplest conceptual model can be useful in communicating and enhancing understanding of the ramifications of uncertainty (Henrichs et al. 2010; Milner-Gulland and Shea 2017). Model simplicity is also desirable in decision-making for transparency, ease of validation, and description of the models (Caro et al. 2012). An important aspect in the development and operationalization of scenarios will be to translate them in a way that allows end users (policy makers, civil society organizations among others) to incorporate them into their decision-making. This can either be done by systematically co-designing scenarios with stakeholders, or by translating existing scenarios into a commonly understandable language.

### Focus on social equity, power relations, and discourses

An unequal distribution of benefits derived from natural assets has important implications for human well-being and social equity (Berbés-Blázquez et al. 2016). Recently, Schröter et al. (2017) provided a framework to link ecosystem services to sustainable development through strategies to achieve sustained provision of ecosystem services. These include strategies for the equitable intra- and inter-generational distribution of ecosystem services. Although central to the United Nation’s SDGs, the assessment of how ecosystem services benefits and values are distributed has not frequently been addressed in the sustainability literature (Boerema et al. 2016). Social equity is about recognition of multiple value systems, effective participation in decision-making, and just/fair distribution of benefits and burdens (Pascual et al. 2014). Social equity in the distribution of benefits must be addressed in future efforts to respond and contribute to the achievement of the SDGs such as the promotion of peaceful and inclusive societies (SDG 16), ending poverty (SDG 1), and promoting protection and restoration of ecosystems (SDG 15), and to better target the development of capacity building towards achieving sustainability (SDG 17) (Griggs et al. 2013). Incorporating the assessment of fairness in the distribution of services and benefits among social groups is urgently needed, as the concept is increasingly adopted to address issues relating to poverty and vulnerability. Further, as inequity is often seen as a source of conflict, prioritizing equity and fairness in the access to ecosystem services and benefits can facilitate acceptance and subsequent higher likelihood policy uptake (Halpern et al. 2013). Addressing these dimensions will steer science and policy towards targeting ecosystem services management for achieving sustainability and social justice.

Imbalances of power are a relevant variable determining access, use, and distribution of natural assets. This is challenging, as these imbalances result from interactions between multiple factors such as political, ecological, and socioeconomic (Hicks and Cinner 2014; Pascual et al. 2014). For example, many South American landscapes are intensively managed for intensive agricultural use—such as soybean (in Brazil and Argentina), banana (in Ecuador), and avocado (in Mexico)—that are often surrounded by poor and marginalized communities. These agricultural goods are often then traded in the market benefiting stakeholders who are often disconnected from the local human-environmental context of these places (Berbés-Blázquez et al. 2016). Future natural assets research should focus on addressing power imbalances across actors to deliver socially fairer outcomes and more equitable access to natural assets (Pascual et al. 2017).

Understanding how different actors exercise power through their discourses is one of the critical mechanisms for the knowledge of natural assets to be tailored to local realities. According to Dryzek (1997), a discourse is:

‘‘A shared way of apprehending the world. Embedded in language, it enables those who subscribe to it to interpret bits of information and put them together into coherent stories or accounts’’.

Getting the discourse right is critical for achieving natural assets sustainability, as this can provide a narrative through which individuals and communities can validate and initiate actions, addressing issues of agency and empowerment which are important for framing relations with natural assets (Rose 1990; Fortmann et al. 1995; McHenry 1996; Gelcich et al. 2005). Stakeholders are considered to be actively involved in the production of discourses, which are then used to give meaning to social–ecological phenomena (Fortmann 1990; Hajer 1995). Local discourses are important as a way of legitimizing worldviews and positioning actors in relation to natural assets (Rose 1990; Fortmann et al. 1995; Gelcich et al. 2005). In doing so they allow incentives and dominance of particular sets of values to be addressed when extending the natural assets concept to real-world applications.

## Conclusion

There is a momentum for the implementation of the natural assets approach, as several international initiatives are focusing attention on the recognition of human–nature relationships for human well-being. The challenge is to turn this recognition into policies and decisions that can guide the sustainable management of natural assets. The natural assets approach could play a key role by emphasizing the role of human actions and aiming to connect epistemologies and knowledge actors engaged in management and conservation. However, this potential will remain unrealized in the absence of an implementation pathway that addresses the inherent challenges of turning knowledge into actions. Our clarification of the natural assets framing and its underlying concepts coupled with the need to translate and bridge among different knowledge systems and different perspectives on people’s relationships with nature provides such a pathway. The solutions are outlined as follows: embracing richer collaborative decision processes towards sustainability to improve environmental decision-making; focusing on the development of scenarios capturing social and ecological interactions and focusing on social equity, power relationships, and discourses to guide natural assets decision-making for more socially fair outcomes. These insights can be used to inform and prioritize future research facilitated under the Natural Assets KAN.

## Electronic supplementary material

Below is the link to the electronic supplementary material.

Supplementary material 1 (DOCX 12 kb)
