# Supplementary material for: Framing natural assets for advancing sustainability research: translating different perspectives into actions
Source: Sustain Sci. 2018 Jul 17;13(6):1519–31. doi: 10.1007/s11625-018-0599-5 (PMC6267164; doi:10.1007/s11625-018-0599-5)
Supplement: Supplementary file 1 — Supplementary material 1 (DOCX 12 kb) [file 11625_2018_599_MOESM1_ESM.docx]

**Supplementary Material**

We looked in the Thomson Reuters Web of Science database using the following conditions:

1. DOCUMENT TYPES: (Article OR Review)

2. Languages: All languages

3. Timespan=All years

4. Indexes=SCI-EXPANDED, SSCI, A&HCI

For the search terms we looked for “ecosystem service*" resulting in 13752 references and "natural capital*” resulting in 910 references, that were included in the topic analysis. A topic analysis is a simplified representation of a collection of documents. Topic analysis software identifies words with topic labels, such that words that often show up in the same document are more likely to receive the same label. It can identify common subjects in a collection of documents – clusters of words that have similar meanings and associations – and discourse trends over time and across geographical boundaries. The approach chosen is to identify these topics and to use them to cluster the articles without prejudice of their origin. This will enable us to identify definitions for each cluster / topic where we would expect common concepts for the term "ecosystem service" and “natural capital”.

The topic analysis was done using the program Topic Modelling Tool (TMT). This program is a graphical user interface for the command line program MALLET (McCallum, 2002). TMT returns the code used to turn MALLET, so we can easily write a script do that the whole process does not rely on a GUI tool anymore and is reproducible. As the text corpus for each paper, we used the fields title and abstract from both search terms separately.

**References**

McCallum, A. K. (2002). MALLET: A Machine Learning for Language Toolkit . Retrieved from http://mallet.cs.umass.edu/index.php
